# Association between parity and bone mineral density in postmenopausal women

**DOI:** 10.1186/s12905-022-01662-9

**Published:** 2022-03-23

**Authors:** Yimei Yang, Shanshan Wang, Hui Cong

**Affiliations:** 1 Department of Obstetrics and Gynecology, Affiliated Hospital of Nantong University, Nantong, 226001 China; 2 Department of Blood Transfusion, Affiliated Hospital of Nantong University, Nantong, 226001 China; 3 Departments of Laboratory Medicine, Affiliated Hospital of Nantong University, Nantong, 226001 China

**Keywords:** Parity, Bone mineral density, Postmenopausal women, Osteoporosis, NHANES

## Abstract

**Background:**

Pregnancy has been considered a risk factor for the development of osteoporosis. Despite much research in this field, the relationship between parity and bone mineral density (BMD) is still controversial. Therefore, we conducted this study to investigate whether there was an association between parity and BMD of the femoral neck and lumbar spine in postmenopausal women.

**Methods:**

Cross-sectional study was conducted using data from the National Health and Nutrition Examination Survey (NHANES). Three linear regression models, Model 1 (unadjusted), Model 2 (adjusted for age and body mass index (BMI)), and Model 3 (adjusted for all covariates), were established to evaluate the relationship between parity and BMD. In addition, the *p value* trend of BMD in the different parity groups was mutually verified with the results of multiple regression. Multiple logistic regression models were used to assess the relationship between parity and osteoporosis.

**Results:**

In total, 924 postmenopausal women aged 45–65 years were eligible for this study. After adjustment for potential confounders, women with ≥ 6 parities had significantly lower lumbar spine BMD than women with 1–2 parities (*β* = − 0.072, 95% CI: − 0.125, − 0.018, *P* = 0.009). However, there was no correlation between parity and femoral neck BMD in any of the three regression models. Furthermore, ≥ 6 parities were associated with a significantly higher prevalence of lumbar spine osteoporosis compared with 1–2 parities (*OR* = 3.876, 95% CI: 1.637, 9.175, *P* = 0.002).

**Conclusions:**

After adjustment for BMD-related risk factors, ≥ 6 parities were associated with decreased lumbar spine BMD but not femoral neck BMD in postmenopausal women. This suggests that postmenopausal women with high parity are at increased risk of lumbar osteoporotic fractures and should pay more attention to their bone health.

## Background

Osteoporosis is one of the most common chronic metabolic skeletal diseases, increasing the risk of bone fragility and fracture due to low bone mass and the destruction of bone microstructure [1]. Postmenopausal women are at high risk for osteoporosis because estrogen deficiency accelerates bone turnover with net bone loss [2]. A study from the National Health and Nutrition Examination Survey (NHANES) showed that among older US adults, the prevalence of osteoporosis and low bone mass in women was significantly higher than that in men, whether at the femoral neck or lumbar spine [3]. Bone mineral density (BMD), as an index to evaluate the mineral content in bone, is often used in the diagnosis of osteoporosis. Low BMD is strongly related to an increased risk of fracture, which increases the incidence rate and mortality for elderly women [4]. Therefore, it is very important to determine the possible risk factors for low BMD in postmenopausal women.

Pregnancy has been considered a risk factor for the development of osteoporosis [5, 6]. Theoretically, bone mass may decrease due to calcium requirements during pregnancy, while on the contrary, bone mass may increase due to higher estrogen levels in the third trimester of pregnancy and increased bone load caused by weight gain during pregnancy [5, 6]. Despite much research in this field, the relationship between parity and BMD is still controversial. Therefore, we conducted a cross-sectional study to investigate whether there was an association between parity and BMD of the femoral neck and lumbar spine in postmenopausal women.

## Method

### Study population

This study was conducted using data from the NHANES, a two-year-cycle cross-sectional survey conducted by the Centers for Disease Control and Prevention (CDC). Participants in each NHANES cycle were identified through stratified, multi-stage probability sampling of the non-institutionalized US population. The data from this survey have been widely used in epidemiological research, nutritional status assessments, and disease risk factor investigations [7–10]. The Ethics Review Board of the National Center for Health Statistics (NCHS) approved the survey protocols and written informed consent was obtained from all participants.

**Fig. 1:**
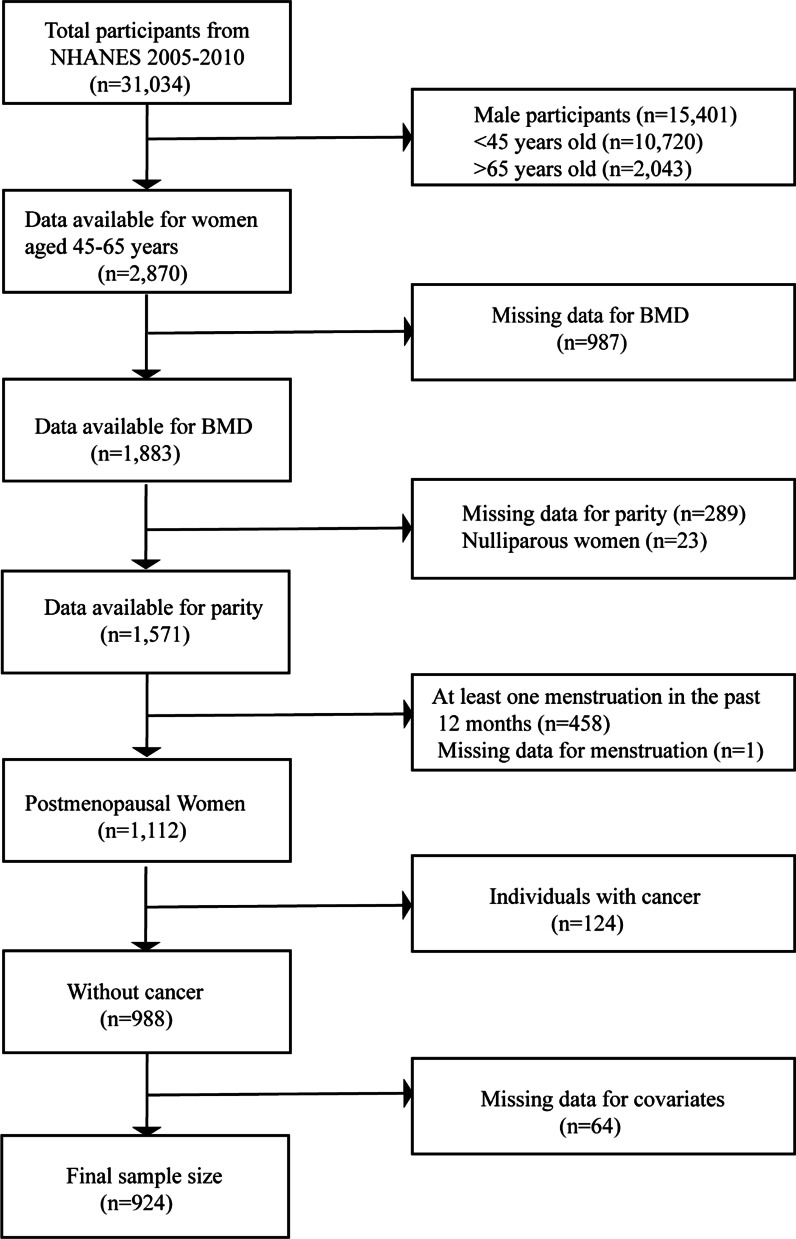
Flow chart of research sample selection

In this study, we merged the data of the three cycles (2005–2006, 2007–2008, and 2009–2010). From 2005 to 2010, a total of 31,034 individuals participated in the NHANES project. Among 2870 women aged 45–65 years, we excluded participants with missing BMD (n = 987) and parity (n = 289) data and women who had not given birth (n = 23). Among 1112 postmenopausal women, participants with cancer (n = 124) and missing covariate data (n = 64) were further excluded. Those who answered “yes” to the question “Have you ever been told by a doctor or other health professional that you had cancer or a malignancy of any kind?” were defined as cancer participants. Finally, 924 postmenopausal women aged 45–65 years were included in our study (Fig. 1).

## Study variables

The independent variable of our study was parity. Parity information was obtained from the question of reproductive health in the module of questionnaire data: “How many deliveries live birth result?” We divided parity into three groups: 1–2, 3–5 and ≥ 6, of which the 1–2 parity group was the reference group.

The dependent variables in our study were the femoral neck and lumbar spine BMD. From 2005 to 2010, dual-energy X-ray absorptiometry (DXA) scans of the proximal femur and lumbar spine were performed at the NHANES mobile examination center (MEC). The radiation exposure from DXA for both the femur or spine scan is extremely low at less than 20 uSv. The DXA examinations were performed by trained and certified radiographers. In addition, we converted BMD to T-scores, and according to established criteria, T-scores ≤ − 2.5 was classified as osteoporosis.

The selection of covariates was based on previous literature [11–13]. Age, family poverty income ratio (PIR), race, education level, smoking behavior, alcohol consumption, physical activity (PA), reproductive health, hormone replacement therapy (HRT), ever treated for osteoporosis, drug (e.g., corticosteroid, anticoagulant, anticonvulsive drug and immunosuppressive) use and disease history were obtained by self-reports. The family PIR was categorized as <1.3 (low income), 1.3–3.5 (middle income), and ≥3.5 (high income). Alcohol consumption was divided into three categories: non-drinkers, moderate drinkers, and heavy drinkers. Female drinkers, on average, have less than 2 drinks per day as moderate drinkers and 2 or more drinks as heavy drinkers [14]. PA was divided into two types according to the metabolic equivalent of task (MET): active and non-active. The total MET-minutes/week was calculated based on participants’ self-reported activity types and time. The MET-minutes/week was calculated by multiplying the MET value of each activity by the total number of minutes of each activity per week. Finally, by summing the MET-minutes/week of each activity, the total MET-minutes/week of all activities was calculated. Participants were defined as non-active with MET-minutes/week < 500 and ≥ 500 as active [15]. Referring to previous study [16], HRT was divided into currently using, previously used and never used. Menopausal status was defined as not having a menstruation in the past 12 months, with the exception of pregnancy and breastfeeding. Years since menopause were calculated by subtracting the age at the last menstrual period from the age at the time of the survey. Ever treated for osteoporosis, drug use and disease history are only shown in the table as “yes”. Body mass index (BMI) was calculated as weight in kilograms divided by height in meters squared. Details for total calcium and serum phosphorus are provided in the standard biochemical profile under the laboratory data section of the NHANES website.

## Statistical analyses

NHANES sample weights were considered in the analysis to represent the noninstitutionalized civilian population of the United States. EmpowerStats statistical software (X&Y Solutions, Boston, MA) and R software (version 3.4.3) were used for all analyses, and a *p* < 0.05 was considered statistically significant.

The data are reported as mean ± SD and Min-Max for continuous variables and percentages for categorical variables. The *p* values of continuous variables and categorical variables were calculated by weighted linear regression model and weighted chi-square test, respectively.

Three linear regression models, Model 1 (unadjusted), Model 2 (adjusted for age and BMI), and Model 3 (adjusted for all the covariates listed in Table 1), were established to evaluate the relationship between parity and BMD of the femoral neck and lumbar spine. In addition, the *p value* trend of BMD in the different parity groups was mutually verified with the results of multiple regression. Multiple logistic regression models were used to assess the relationship between parity and osteoporosis.

**Table 1:** The features of the participants based on parity

Characteristics	Parity			*p* value
	1–2	3–5	≥ 6	
Age (mean ± SD), years	55.40 ± 5.28 (45–65)	56.29 ± 5.68 (45–65)	57.44 ± 5.46 (47–65)	0.020
Race (%)				< 0.001
Non-Hispanic white	77.94	66.57	51.02	
Non-Hispanic black	9.47	11.94	16.48	
Mexican American	2.83	9.80	24.77	
Other race	9.76	11.70	7.73	
Education level (%)				0.004
Less than high school	13.08	18.30	37.54	
High school	28.67	28.30	33.78	
More than high school	58.25	53.40	28.68	
Family PIR (%)				< 0.001
< 1.3	11.20	17.57	43.60	
1.3 –3.5	24.25	33.99	32.89	
≥ 3.5	59.59	41.86	18.99	
Not recorded	4.96	6.58	4.52	
BMI (mean ± SD), kg/m^2^	27.58 ± 6.00 (15.44–50.82)	29.27 ± 6.33 (15.83–53.89)	30.49 ± 6.25 (17.93–41.56)	< 0.001
Total calcium (mean ± SD), mmol/L	2.38 ± 0.09 (2.125–2.850)	2.38 ± 0.09 (2.175–2.825)	2.40 ± 0.08 (2.100-2.675)	0.444
Serum phosphorus (mean ± SD), mmol/L	1.27 ± 0.16 (0.807–1.841)	1.27 ± 0.17 (0.807–2.163)	1.27 ± 0.12 (0.969–1.679)	0.813
Age at menarche (mean ± SD), years	12.71 ± 1.72 (8–19)	12.95 ± 1.55 (8–19)	12.61 ± 1.75 (6–18)	0.097
Years since menopause (mean ± SD), years	9.69 ± 7.83 (1–45)	11.27 ± 8.90 (1–42)	9.32 ± 6.96 (1–30)	0.019
Smoking behavior (%)				0.505
Current	19.20	20.30	27.71	
Former	28.14	24.23	16.62	
Never	52.66	55.46	55.67	
Alcohol consumption (%)				< 0.001
Non-drinker	17.60	27.95	41.64	
Moderate drinker	47.36	45.55	48.17	
Heavy drinker	35.04	26.50	10.19	
PA (%)				0.086
Non-active	35.37	42.77	40.28	
Active	64.63	57.23	59.72	
HRT (%)				0.007
Currently using	12.44	7.16	1.05	
Previously used	32.16	35.92	15.32	
Never used	55.40	56.92	83.63	
Ever treated for osteoporosis (%)	9.39	7.44	1.04	0.250
Drug use (%)	9.27	7.28	4.71	0.470
Diabetes (%)	8.05	11.67	17.92	0.160
Arthritis (%)	34.43	42.13	45.07	0.053
Emphysema (%)	1.15	1.64	1.55	0.820
Chronic bronchitis (%)	5.55	8.56	13.25	0.102
Liver disease (%)	3.89	3.23	0.00	0.558
Kidney disease (%)	2.29	1.99	2.84	0.935
Thyroid disease (%)	19.61	19.58	14.40	0.818

Mean ± SD and Min–Max for continuous variables and % for categorical variables

## Result

In total, 924 postmenopausal women aged 45–65 years were eligible for this study (Fig. 1). The features of the participants based on parity are shown in Table 1. There were significant differences in age, BMI, years since menopause, family PIR, race, education level, HRT, and alcohol consumption among the different parity groups (1–2; 3–5; ≥ 6).

Table 2 shows the associations between parity and femoral neck BMD. There was no correlation between parity and femoral neck BMD in any of the three regression models (Table 2).

**Table 2:** Associations between parity and femoral neck BMD

Parity	Model 1	Model 2	Model 3
	*β* (95% CI) *p* value	*β* (95% CI) *p* value	*β* (95% CI) *p* value
1–2	Reference	Reference	Reference
3–5	0.010 (− 0.007, 0.027) 0.231	− 0.003 (− 0.017, 0.012) 0.738	− 0.003 (− 0.018 , 0.011) 0.670
≥ 6	0.022 (− 0.029, 0.074) 0.395	0.002 (− 0.043, 0.047) 0.928	0.000 (− 0.044, 0.044) 0.998

Model 1: no covariates were adjusted

Model 2: age and BMI were adjusted

Model 3: age, BMI, total calcium, serum phosphorus, age at menarche, years since menopause, family PIR, race, education level, smoking behavior, alcohol consumption, PA, HRT, ever treated for osteoporosis, drug use, diabetes, arthritis, emphysema, chronic bronchitis, liver disease, kidney disease, and thyroid disease were adjusted

Table 3 shows the associations between parity and lumbar spine BMD. After adjustment for potential confounders, ≥ 6 parities had significantly lower lumbar spine BMD than 1–2 parities (*β* = − 0.072, 95% CI: − 0.125, − 0.018, *P* = 0.009) (Table 3). This significant trend of lumbar spine BMD was further verified and is shown in Table 4.

**Table 3:** Associations between parity and lumbar spine BMD

Parity	Model 1	Model 2	Model 3
	*β* (95% CI) *p* value	*β* (95% CI) *p* value	*β* (95% CI) *p* value
1–2	Reference	Reference	Reference
3–5	− 0.006 (− 0.025, 0.014) 0.560	− 0.016 (− 0.034, 0.002) 0.080	− 0.014 (− 0.031, 0.004) 0.130
≥ 6	− 0.062 (− 0.120, − 0.003) 0.039	− 0.078 (− 0.132, − 0.023) 0.005	− 0.072 (− 0.125, − 0.018) 0.009

Model 1: no covariates were adjusted

Model 2: age and BMI were adjusted

Model 3: age, BMI, total calcium, serum phosphorus, age at menarche, years since menopause, family PIR, race, education level, smoking behavior, alcohol consumption, PA, HRT, ever treated for osteoporosis, drug use, diabetes, arthritis, emphysema, chronic bronchitis, liver disease, kidney disease, and thyroid disease were adjusted

**Table 4:** Trend of femoral neck and lumbar spine BMD based on parity

Parity	Sample size (*n*)	Adjust Mean (95% CI)	
		Femoral neck BMD (g/cm^2^ )	Lumbar spine BMD (g/cm^2^ )
1–2	462	0.763 (0.752, 0.774)	0.964 (0.951, 0.978)
3–5	409	0.760 (0.748, 0.772)	0.950 (0.936, 0.965)
≥ 6	53	0.763 (0.721, 0.805)	0.892 (0.841, 0.943)
P for trend		0.755	0.010

Age, BMI, total calcium, serum phosphorus, age at menarche, years since menopause, family PIR, race, education level, smoking behavior, alcohol consumption, PA, HRT, ever treated for osteoporosis, drug use, diabetes, arthritis, emphysema, chronic bronchitis, liver disease, kidney disease, and thyroid disease were adjusted

Table 5 shows the associations between parity and osteoporosis of femoral neck and lumbar spine. After adjustment for potential confounders, ≥ 6 parities were associated with a significantly higher prevalence of lumbar spine osteoporosis compared with 1–2 parities (*OR* = 3.876 , 95% CI: 1.637, 9.175, *P* = 0.002).

**Table 5:** Associations between parity and osteoporosis of femoral neck and lumbar spine

Parity	Osteoporosis (*n*)	No osteoporosis (*n*)	Odds ratio (95% CI)	*p* value
Femoral neck				
1–2	20	442	Reference	—
3–5	16	393	0.974 (0.411, 2.307)	0.952
≥ 6	1	52	1.019 (0.099, 10.488)	0.987
Lumbar spine				
1–2	34	428	Reference	—
3–5	43	366	1.096 (0.633, 1.900)	0.743
≥ 6	17	36	3.876 (1.637, 9.175)	0.002

Age, BMI, total calcium, serum phosphorus, age at menarche, years since menopause, family PIR, race, education level, smoking behavior, alcohol consumption, PA, HRT, ever treated for osteoporosis, drug use, diabetes, arthritis, emphysema, chronic bronchitis, liver disease, kidney disease, and thyroid disease were adjusted

## Discussion

This study investigated the relationship between parity and BMD in postmenopausal women aged 45–65 years. In our study, we divided parity into three groups and characterized the study population accordingly. Our results showed that parity was negatively correlated with lumbar spine BMD in all three regression models, but not with femoral neck BMD. These results were verified by the P for trend of BMD based on parity. Consistent with the above results, the prevalence of lumbar spine osteoporosis was significantly higher in the highest parity group.

Previous research has reported conflicting conclusions. A study of postmenopausal women in Morocco showed results that were consistent with ours. The authors found that patients with 6 or more parities had significantly lower lumbar spine BMD values than patients with other numbers of parities, but there was no significant difference in the femoral neck BMD values [17]. Gur et al. [18] also found that there was a significant negative correlation between the number of pregnancies and spine BMD but no significant correlation with the femoral neck BMD. Heidari et al. [19] had similar findings. They reported an independent association between parity and lumbar spine osteoporosis but not for the femoral neck and a 13% increased risk of lumbar spine osteoporosis per parity. In addition, Demir et al. [20] and Seo et al. [11] observed that high parity was a risk factor for low BMD in postmenopausal women. However, other studies have shown no relationship between parity and BMD or osteoporosis [21, 22], while another study suggested a protective effect of high parity on postmenopausal osteoporosis [23]. The results of these conflicts may be due to different ethnic groups, lifestyles, nutritional status and so on.

Calcium is in high demand during pregnancy and breastfeeding due to the growth of fetal and newborn bones. The physiological requirement of a full-term singleton for calcium is about 30 g [24]. If the mother’s bone minerals are the only source of calcium, the mother’s bones will lose approximately 3% (30 g/1000 g) of the mineral with each pregnancy [18]. However, there are some adaptive changes during pregnancy and breastfeeding. During pregnancy, the efficiency of intestinal calcium absorption doubles to meet the need of the fetus for calcium, while during lactation, skeletal resorption increases to provide calcium for milk production. These hormone-mediated adaptations usually meet the daily mineral needs of the fetus and infant without adverse long-term effects on the maternal bones [25]. Fokter et al. [26] reported a case of proximal femoral fracture caused by osteoporosis in the third trimester of first pregnancy, with excellent healing effect after surgery, suggesting that transient osteopenia during pregnancy has the potential for normal healing. However, it is not clear whether the bone loss from multiple pregnancies is fully compensated. Animal studies have shown that as the number of litters increases, the decrease in trabecular bone density becomes irreversible [27, 28]. Therefore, when the number of parities increases, bone loss may not be fully recovered [29]. The mechanisms underlying the effects of parity on the skeleton are complex, and more basic and clinical studies are needed in the future to clarify this relationship.

In our study, we found differences in the influence of parity on lumbar spine and femoral neck BMD. This may be due to the difference in bone structure between the lumbar spine, which is dominated by trabecular bone, and the femoral neck, which is dominated by cortical bone. The increased calcium demand during pregnancy and lactation leads to increased bone absorption. Due to the larger surface area of bone trabeculae, osteoclasts reabsorb more rapidly [13]. MicroCT studies have confirmed that calcium restriction leads to greater reductions in bone volumes, trabecular number and thickness, and tissue density [30]. These results support our finding that parity is negatively correlated with lumbar spine BMD.

In this study, we designed three models to observe the correlation between parity and BMD by adjusting for different confounding factors. However, there are some limitations to this study. First, although the questionnaire data were obtained from face-to-face interviews, there were still some recall biases. Second, the disease history was based on self-reports and was not cross-checked with medical records. Third, due to lack of reproductive information such as twins, abortions, stillbirths, and breastfeeding duration, the impact of these factors on BMD was not considered. Finally, although we combined data from three cycles, the number of eligible nulliparous women was too small to be included from the analysis, so we did not compare the BMD values of nulliparous women with those of parous women. Future studies with a larger sample size and involving nulliparous women are needed.

## Conclusions

After adjustment for BMD-related risk factors, ≥ 6 parities were associated with decreased lumbar spine BMD but not femoral neck BMD in postmenopausal women. This suggests that postmenopausal women with high parity are at increased risk of lumbar osteoporotic fractures and should pay more attention to their bone health.

## Data Availability

All the data are publicly available online (https://www.cdc.gov/nchs/nhanes/).

## Abbreviations

BMD: Bone mineral density
BMI: Body mass index
NHANES: National health and nutrition examination survey
CDC: Centers for disease control and prevention
NCHS: National center for health statistics
DXA: Dual-energy X-ray absorptiometry
MEC: Mobile examination center
PIR: Poverty income ratio
PA: Physical activity
HRT: Hormone replacement therapy
MET: Metabolic equivalent of task
